# Adaptation of the Beverage Intake Questionnaire-15 (BEVQ-15) into Turkish: validity and reproducibility study

**DOI:** 10.1186/s12937-025-01169-7

**Published:** 2025-10-07

**Authors:** Sabriye Arslan, Selin Keskin, Valisa Hedrick, Feride Ayyıldız

**Affiliations:** 1 Department of Nutrition and Dietetics, Faculty of Health Sciences, Gazi University, Ankara, 06490 Türkiye; 2 Department of Human Nutrition, Foods and Exercise, Virginia Tech, 229 C Wallace Hall (0430), Blacksburg, VA 24061 USA

**Keywords:** Beverages, Food frequency questionnaire, Dietary assessment, Validity, Reproducibility

## Abstract

**Purpose:**

This study aimed to translate and culturally adapt the Beverage Intake Questionnaire (BEVQ-15) to the Turkish population and to assess its validity and reproducibillity.

**Methods:**

This cross-sectional study included adults and older residing in Ankara, Turkey. The study process involved the translation of the BEVQ-15 from English into Turkish and its adaptation to the Turkish community. Adaptations to the original BEVQ-15 included separating the black tea and coffee category into distict categories, as well as herbal tea. Additionally, plain mineral water, flavored mineral water, kefir, and turnip juice were presented as individual categories. The adaptation of BEVQ-15 to Turkish preferences resulted in the BEVQ-21. Participants come to three visits, each two weeks apart. The BEVQ-21 was administered at visits 1 and 3, and a three day dietary record was returned during visit 2.. The BEVQ-21 was conducted at visit one (BEVQ-1) and visit three (BEVQ-2). Validity and reproducability statistical analyses were conducted using Wilcoxon signed-rank tests, Bland–Altman plots, and Spearman correlations.

**Results:**

Fifty-one participants completed all study visits. Minimal yet significant differences were identified between the two assessment tools (BEVQ-2 and DR) across various beverage categories, with mean differences ranging from 3 to 82 mL and 0 to 16 kcal. According to Bland–Altman plots between BEVQ-21 and dietary records, differences for water (mL), regular mineral water (mL), whole and flavored milk (mL and kcal), soft drinks (mL and kcal), black tea (mL), herbal teas (mL), and total beverage intake (mL) were found to be approximately consistent within the boundaries (*p* < 0.05). For reproducibility, sugary beverage and total beverage consumption were significantly associated between the first and second administration of the BEVQ-21 (*r* = 0.44–0.65, *p* ≤ 0.05).

**Conclusions:**

The adapted BEVQ-21 for the Turkish population demonstrated validity and reproducibility for most types of beverage intake among adults in Turkey.

## Introduction

Obesity has emerged as a significant global health issue, posing a risk for chronic noncommunicable diseases such as diabetes and cardiovascular diseases [1]. Obesity is affected by many factors such as age, genetics and environmental factors [2]. Nutrition, one of the leading factors, contributes to obesity due to the increase in energy intake and unbalanced intake of nutrients as compared to needs [3]. The increase in energy intake not only from food but also from beverages contributes to the increase in body weight [4]. Therefore, it is very important to evaluate beverage consumption when evaluating dietary intake. The worldwide increase in obesity prevalence has led to scientific investigations into the relationship between body weight and the consumption of sugary beverages [5]. Scientific research suggests that one of the contributing factors to excess energy intake is the overconsumption of sugary drinks [6]. Global assessments of sugar intake indicate a noticeable rise in the consumption of sugar-sweetened beverages [7]. The World Health Organization emphasizes that the energy derived from added sugars should not exceed 10% of the total daily energy intake and ideally aims to reduce this percentage to below 5%, as reduced consumption of sugary drinks is associated with a reduced risk of obesity.

Assessment of beverage intake is important not only for determining contribution to energy intake, but also for hydration status [8] which is associated with the maintenance of physical and mental functions [9]. Assessing past dietary habits through a food or beverage frequency questionnaire is useful for identifying risk factors for obesity [10]. Surveys used for food and beverage consumption not only provide convenience to participants and researchers but also contribute to evaluating whether the quantity of consumed foods or beverages is appropriate over a specific period [11]. Surveys also facilitate the assessment of non-daily consumed food items and total fluid intake since they allow participants to evaluate their intake over a long-term period vs acute intake from food diaries/recalls [12]. Considering the crucial role of beverages in the diet and their impact on health and disease, beverage intake must be accurately assessed. Beverage Intake Questionnaire (BEVQ), a tool developed for this purpose, was originally designed for use in Western culture and targeted at adults. However, over time, adaptation studies have been conducted for different cultural contexts, such as French, Arabic, and Spanish, as well as for various age groups, including children, adolescents, and adults [13–18].

In this study, the Turkish adaptation of the Beverage Intake Questionnaire-15 (BEVQ-15), a beverage frequency questionnaire, was examined for validity and reproducibility. The BEVQ-15 was designed to rapidly assess beverage consumption in adults, including the average daily intake of sugary beverages across 15 categories [19, 20]. The questionnaire includes questions that inquire about how often and how much 15 types of beverages are consumed. In 2020, the BEVQ-15 was updated to match current dietary patterns and trends [21]. In order to adequately reflect current beverage consumption trends and increase its applicability to other populations with high fluid intake, such as athletes, some updates have been made to the BEVQ-15. For example, commonly consumed nut milks and sports drinks have been added, and the limited ceiling threshold of 60 fl oz has been removed for all beverage categories [21]. As major changes were made for the updated BEVQ-15, and no validity or reproducibility studies have been conducted in Turkey for the updated BEVQ-15, this study was designed to fill this gap. We believe that our study is important in terms of evaluating the suitability of this update, which was made depending on the changes in literature and living conditions, to Turkish culture. While making this adaptation, it is very valuable in terms of cultural adaptation to add some beverages frequently consumed in Turkish culture to BEVQ. For example, our country is among the countries with the highest tea consumption in the world [22]. One of Turkey’s traditional beverages, turnip juice, is widely consumed, especially in the southern part of our country, and its production and consumption are increasing in cities such as Antakya, Maraş, Istanbul and Ankara. In recent years, it has also taken its place in the markets of European cities with a large Turkish population [23]. The consumption of kefir, a traditional Caucasian and Middle Eastern drink, has increased in our country in recent years [24]. In our country, which has great potential in terms of natural mineral water resources, the consumption of mineral water has also increased with the introduction of fruit-flavored mineral waters into the market [25]. The adaptation of this updated questionnaire to Turkish culture, along with a validity and reproducibility study, will contribute to the literature, particularly in studies investigating the relationship between obesity and hydration, as well as beverage intake, within a Turkish population.

## Methods

### Subjects and design

This cross-sectional study included adults aged greater than 18 years old who resided in the Ankara province. Individuals with taste disorders, eating disorders, or hydration imbalances were excluded from the study, as their beverage consumption frequency may differ. Future studies are recommended to evaluate the validity of the BEVQ in individuals with specific health conditions such as kidney disease, eating disorders, and Sjögren’s syndrome. Individuals without any chronic illnesses were included in the study between December 2022-May 2023. The study protocol was approved by Gazi University Ethic Comisson with code 2022–1277.

**Fig. 1:**
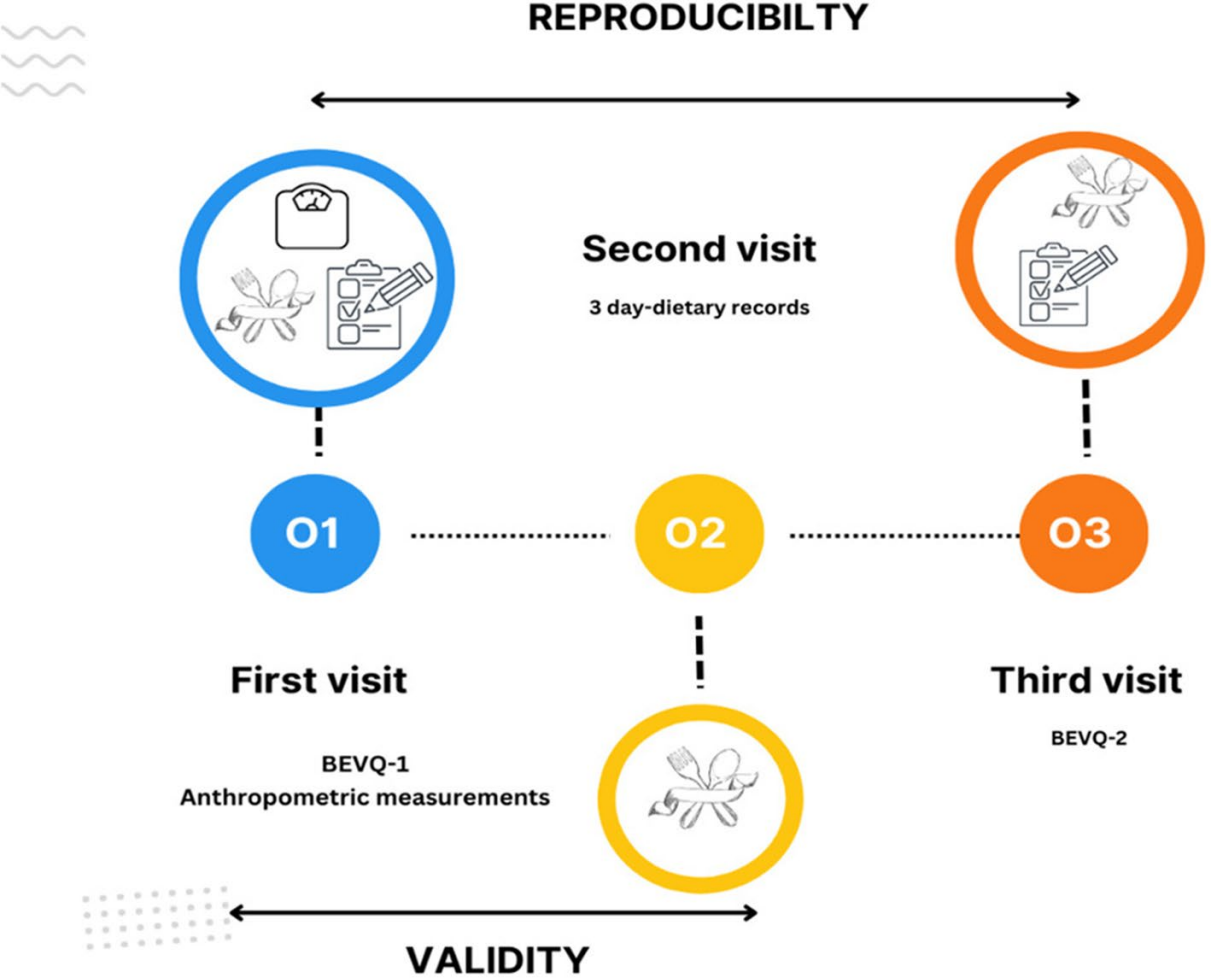
Design of the BEVQ-21 validity and reproducibility study

Participants completed three visits to the laboratory, with each visit occurring at a 2-week interval. At the first visit, the height of the participants was measured using a stadiometer in accordance with the technique [26], and body weight and body composition were taken with Tanita BC-418. For bioelectrical impedance analysis (BIA), participants were instructed to refrain from engaging in intense physical activity 24–48 h prior to the measurement, to arrive in a fasting state (at least 4 h of fasting) without having breakfast, to avoid alcohol consumption within 24 h before the measurement, and to limit excessive fluid intake (such as tea and coffee) at least 4 h prior to the measurement. Additionally, care was taken to ensure that participants did not have any metal objects on their bodies during the measurement [27]. Height measurements were taken using a stadiometer in the Frankfurt plane [26]. During this visit, information about demographic characteristics and health conditions was obtained from the participants, and they completed the BEVQ (BEVQ-1). Participants were given instructions to keep a 3-day dietary record (DR). A 3-day dietary record was used as a reference to evaluate the validity of the beverage consumption questionnaire. To capture both weekend and weekday eating habits, dietary records were kept on two weekdays and one weekend. While collecting 3-day dietary records from individuals, a pictorial food catalog was used to demonstrate portion sizes with measuring tools and photographs, enabling participants to select the appropriate portion size [28]. Dietary records were returned and reviewed for completeness at visit two and analyzed using nutritional analysis software (BEBIS). At visit three, participants filled out the BEVQ again (BEVQ-2) (Fig. 1). For all analyses, the mean beverage intake from the 3-day DR was utilized.

### Adaptation of the BEVQ-15 into Turkish

The updated version of the BEVQ-15 by Fausnacht et al. [21] was used for the Turkish adaptation and validity-reproducibilty study. The translation of the BEVQ-15 was carried out according to the guidelines established by Beaton and colleagues [29]. The original English version of the questionnaire was translated into Turkish by two independent translators proficient in both Turkish and English. One translator had a medical or clinical background, while the other did not. The translations of the two translators were evaluated, and a single form was created. The latest version was retranslated into English by a different bilingual professional. A third native verified that the back-translated version was both conceptually and linguistically akin to the original BEVQ. The BEVQ Turkish questionnaire was completed and subsequently administered to 30 Turkish individuals to assess intelligibility.

To adapt beverage intake habits for the Turkish culture, the content of the survey was modified to fit local customs and traditions. During this stage, the survey's content and questions were revised to carry meaning and relevance for the Turkish community. To assess the adaptation of the survey to Turkish culture, feedback was obtained from experts in the field and potential users based on the results of a pilot study. This feedback was utilized to enhance the survey's clarity and usability. Upon examining the open-ended 'other' option in the questionnaire, it was observed that individuals indicated traditionally consumed turnip juice, kefir, and herbal teas in Turkish culture. Additionally, black tea, frequently consumed in Turkish culture, was included as a separate item independent of coffee, as were plain and flavored mineral water. These modifications resulted in the creation of the BEVQ-21, tailored to Turkish culture. Furthermore, market research has been conducted for the beverages included in the questionnaire, considering that energy differences may vary from country to country. The energy content of beverages in the versions used in Turkey was determined for each beverage by taking the average of the energy content of at least three brands. Although "oz" was used as the volume measure in the original study, mL values were provided instead of oz due to the use of mL as the volume measure in Turkey.

### Statistical analysis

Statistical analyses were performed using SPSS version 24.0. Descriptive statistics for beverage variables are reported as mean ± standard deviation (SD). For beverage categories, total sugary beverages, and total beverages, mean and standard deviation values are reported from the first and second application of the BEVQ (BEVQ-1 and BEVQ-2), and data obtained from the DR in mL and kcal. The sugary-beverage category was calculated from the sum of the following categories: Flavored mineral water, %100 Fruit juice, sweetenedd fruit drink, regular soft drink, energy and sport drink, sweet tea, black tea (with sugar), coffee (with sugar), herbal tea (with sugaar). Since the total beverage mL and energy from the BEVQ-21 and DR did not have a normal distributions non-parametric tests were used.

To assess the validity of BEVQ-21, BEVQ-2 responses for each beverage category were compared to data obtained from DR using the Wilcoxon test. As BEVQ-21 assessed the beverage intake in the last month, BEVQ-2, covering the same time frame, was used to assess validity. For assessing test–retest reproducibility, responses obtained from both applications of BEVQ-21 (BEVQ-1 and BEVQ-2) were compared using the Wilcoxon test.

Relationships between beverage intake variables in terms of reproducibility were evaluated using the Spearman test. The correlation coefficients (r) were classified as small if they were less than 0.29, medium if they ranged from 0.30 to 0.49, and large if they were greater than 0.50 [30]. For agreement between BEVQ-21 and DR, Bland–Altman analyses were performed [31]. An agreement was considered acceptable if 95% of the mean differences were within a range of 2 standard deviations from the mean difference [32, 33].

## Results

Initially, 54 individuals were included in the study; however, due to the inability to reach three individuals for the BEVQ scale evaluated after 15 days (BEVQ-2), the study was completed with 51 participants. The study revealed that 90.2% of the participants were female, 90.2% had university education, and 9.8% had postgraduate education. The mean age (SD) was determined to be 21 (5.1) years.

### Validity

A total of 21 beverage categories plus total sugary beverages and total beverage intake (mL and kcal) were assessed for two assessment tools (BEVQ-2 and DR) (Table 1). Minimal, but significant differences were identified between the two assessment tools (BEVQ-2 and DR) for many of the beverage categories, with mean differences ranging from 3 to 82 mL and 0–16 kcal. Significant mean differences for total sugary beverages and total beverage intake were also found (Table 1).

**Table 1:** Validity and reproducibility of the adapted beverage intake questionnaire for Turkey (BEVQ-21): comparison with dietary records (DR) and results of two applications of BEVQ-21 (BEVQ-1 and BEVQ-2)

Validity^a^				Reproducibility^b^		
Beverage Category	BEVQ-2 Mean (SD)	DR Mean (SD)	Difference^c^ Mean (SE)	BEVQ-1 Mean (SD)	Difference from^c^ Mean (SE)	Correlation *r*
Water						
mL	1611 (592)	1603 (569)	8 (52)	1544 (731)	67 (74)	0.67**
Regular mineral water						
mL	28 (42)	44 (17)	24 (5)**	22 (39)	6 (4)	0,67**
Flavored mineral water						
mL	14 (34)	2 (8)	12 (5)	9 (27)	5 (3)	0.64**
kcal	3 (7)	0.3 (2)	3 (1)	2 (6)	1.0 (0.5)	0.64**
%100 Fruit juice						
mL	18 (44)	8 (22)	10 (7)	11 (27)	7 (6)	0.47**
kcal	9 (21)	3 (8)	6 (3)*	5 (14)	22 (2)	0.47**
Sweetened fruit drink						
mL	12 (29)	4 (13)	8 (33)	8 (28)	4 (5)	0.54**
kcal	6 (15)	3 (9)	3 (3)	44(14)	2 (2)	0.54**
Whole milk or flavored milk						
mL	51 (69)	71 (69)	20 (10)*	54 (69)	3 (7)	0.67**
kcal	27 (38)	42 (40)	16 (7)*	29 (40)	2 (4)	0.71**
Semi-fat or low fat milk, buttermilk or soy milk						
mL	58 (75)	34 (54)	24 (13)	71(79)	14 (10)	0.18
kcal	20 (26)	42(40)	8 (5)	25(27)	4 (4)	0.21
Kefir						
mL	14(26)	7 (30)	7 (36)*	18 (42)	3 (5)	0.76**
kcal	7 (22)	3 (14)	4 (4)	7 (20)	0.5 (3)	0.40**
Nut milk						
mL	20 (102)	2 (9)	18 (14)	5 (18)	14 (14)	0.50**
kcal	4 (21)	0.4 (2)	4 (3)*	1 (4)	3 (3)	0.43**
Regular soft drink						
mL	49 (77)	22 (48)	27 (10)*	37 (64)	13 (8)	0.61**
kcal	18(28)	7 (17)	10 (4)**	13(23)	4 (3)	0.62**
Turnip water						
mL	3(8)	0	3 (1)**	5(19)	1(3)	0.52**
kcal	0.2 (0.4)	0	0.2 (0.1)**	0.2 (1.0)	0.1 (0.1)	0.52**
Energy and sports drinks						
mL	9 (30)	0	9 (4)**	3(24)	5(3)**	0.36**
kcal	3 (11)	0	3 (2)**	1(9)	2 (1)**	0.35*
Diet or artificially flavoured sweetened drinks						
mL	5 (12)	0	5 (2)**	3.4 (24.0)	5.2 (2.7)	0.28*
kcal	0.1 (0.1)	0	0.1 (0)**	1.2 (9)	0.1 (0)	0.20
Sweet tea						
mL	28 (52)	2 (12)	26 (7)**	14 (41)	14 (7)*	0.52**
kcal	5 (9)	0.4 (2)	5 (1)**	3 (8)	3 (1)**	0.57**
Black tea						
mL	269 (312)	187 (221)	82 (34)**	332 (323)	63 (40)	0.52**
kcal	11 (32)	22 (86	11 (13)	7 (30)	4 (5)	0.49**
Coffee (black)						
mL	124 (133)	61 (77)	62 (19)**	129 (167)	5 (26)	0.41**
kcal	5 (10)	6 (19)	2 (3)	2 (6)	3 (2)	0.10
Coffee (with milk/cream)						
mL	101 (205)	21 (41)	80 (30)**	109 (185)	8 (36)	0.20
kcal	17 (49)	4 (12)	13 (7)**	11 (24)	5 (9)	0.04
Herbal tea						
mL	789 (131)	18 (38)	61 (17)**	73 (102)	5 (17)	0.54**
kcal	0.4 (1)	1 (7)	0.7 (1)	0.1 (0.4)	0.2 (0.1)	0.24
Wine						
mL	2 (12)	0	2 (2)	2 (10)	0 (1)	0.49**
kcal	2 (10)	0	2 (1)	2 (8)	0 (1)	0.49**
Hard liquor						
mL	0.3 (2)	0	0.3 (0.3)	0	0.3 (0.3)	-
kcal	0.7 (5)	0	0.7 (0.7)	0.5 (4)	0.1 (1)	0.02
Beer						
mL	0.3 (2)	4 (28)	4 (4)	3 (17)	3 (2)	0.03
kcal	0.1 (1)	0	0.1 (0.1)	1 (7)	1 (1)	0.03
Total sugary beverages						
mL	292 (475)	53 (86)	239 (62)**	195 (265)	97 (66)	0.58**
kcal	68 (111)	17 (24)	52 (15)	42 (57)	26 (14)	0.65**
Total beverage intake						
mL	2495 (883)	2058 (591)	437 (112)**	2457 (919)	38 (123)	0.48**
kcal	137 (111)	81 (46)	56 (21)	114 (102)	23 (18)	0.44**

^a^Validity as determined by comparing the second administration of the BEVQ-21 with the mean reported intake from dietary recalls

^b^Reproducibility as determined by comparing two administrations of the BEVQ-21

^c^Slight differences may be noted from the preceding columns as a result of rounding. These differences reflects comparison between both administrations (BEVQ1-BEVQ2)

*p ≤ 0.05

**p ≤ 0.01

***p ≤ 0.001

**Fig. 2:**
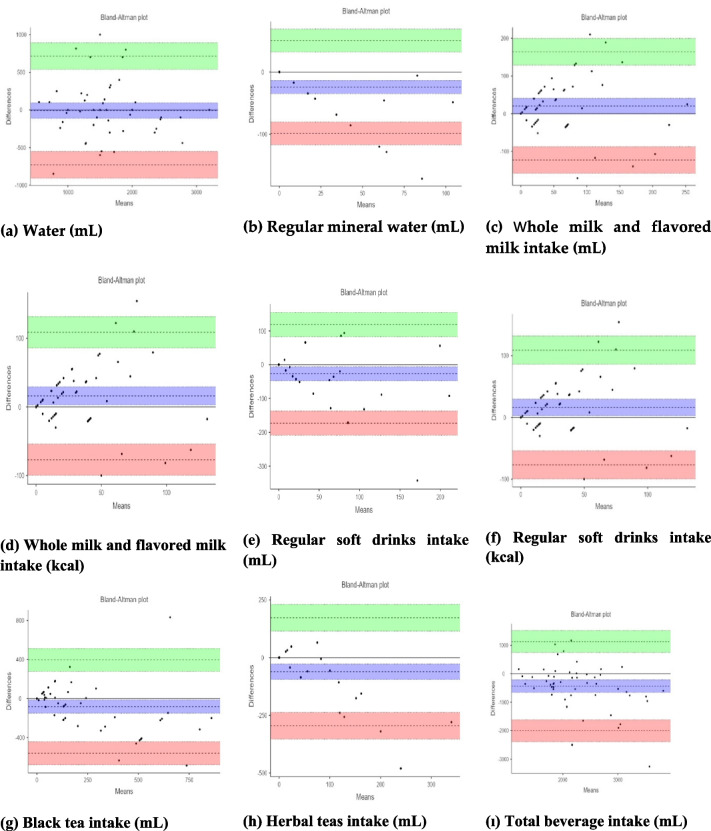
Bland–Altman plots showing the BEVQ-21 (the second administration of the BEVQ was used for this analysis) and dietary intake recalls for (**a**) water (mL); (**b**) regular mineral water (mL); (**c**) whole, flavored milks (mL); (**d**) whole, flavored milks (kcal); (**e**) regular soft drinks (mL); (**f**) regular soft drinks (kcal); (**g**) black tea (mL); (**h**) herbal tea (mL); (**ı**) total beverage (mL) [the centre line represents the mean difference and the upper and lower lines indicate the mean (± 1.96 SD), The values represent the lower and upper limits within the 95% confidence interval]

The Bland–Altman analyses were employed to further assess the agreement level between the BEVQ-21 and DR for total beverage intake (Fig. 2). It can be observed the differences are approximately within the limits of agreement, indicating a reasonable agreement for intake of water (mL), regular mineral water (mL), whole milk and flavored milk (mL and kcal), regular soft drinks (mL and kcal), black tea (mL), herbal teas (mL), total beverage intake (mL). Differences have been identified between DR and BEVQ-21 for beverages other than the specified beverage categories. For total beverage intake (mL), the ICC values were calculated as 0.432 for individual measurements and 0.603 for average measurements (*p* < 0.05).

### Reproducibility

The BEVQ's two applications (BEVQ-1 and BEVQ-2) demonstrated no significant differences between individual beverage categories, total sugary beverages, or total beverage intake, with the exception of energy and sport drinks (mL and kcal) and sweet tea (mL and kcal), which had minimal but significant differences ranging 5–14 mL and 2–3 kcals. Total sugary beverages and total beverage consumption were found to be significantly associated between the first and second applications of BEVQ-21 (*r* = 0.44–0.65, *p* ≤ 0.05) (Table 1).

## Discussion

This research presents the validation and reproducibility of the BEVQ-15, a beverage intake questionnaire, to an updated BEVQ-21 appropriate for the Turkish population. The original scale was modified to create the BEVQ-21 by including several alterations tailored to the Turkish cultural context. While adapting the questionnaire into Turkish, distinct choices were provided for black tea, regular mineral water, flavored mineral water and plain water. Kefir, turnip juice, and herbal teas, which were previously absent in the original scale, have been included in the BEVQ-21. Other versions of the BEVQ-15 have been successfully created through translation and cultural adaptation in a similar fashion [18].

Evaluating the suitability of agreement measurements in comparison to other reference methodologies is challenging [34]. A study conducted to create a FFQ for adolescents to assess their consumption of beverages and snacks showed a moderate to substantial ability to rank the items and minimal differences in reliability [35]. Our investigation reveals that the majority of beverage categories have validity within the medium-large range. The correlation coefficient for flavored mineral water, %100 fruit juice, sweetened fruit drink, semi-fat or low-fat milk, buttermilk or soy milk, nut milk, sweet tea, coffee (with milk/cream), and beer between BEVQ-2 and diet records were small. The BEVQ-21 is anticipated to exhibit slight disparities from dietary records due to its coverage of different distinct time intervals and the reflection of short-term vs long-term intake. Furthermore, the diet records indicated little or not at all consumption of some beverages such as energy and sports drinks (*n* = 0), diet drinks (*n* = 0), turnip water (*n* = 0), wine (*n* = 0), and hard liquor (*n* = 0), beer (*n* = 1), sweet tea (*n* = 2), nut milk (*n* = 2). Therefore, it is thought that the lower correlations may be due to the fact that the beverage categories were consumed less by the participants. There are similar differences in previous studies [15, 36]. A study was conducted to create an online Arabic Beverage Frequency Questionnaire to measure the overall consumption of beverages among Arabic-speaking individuals. The reliability test yielded positive and satisfactory results for most beverages, with the exception of flavored milk and sweetened coffee [15]. Our study's findings align with the Vanderlee et al. study in terms of total alcoholic beverage consumption, sugary drink intake, total beverage volume, and all 17 beverage categories (with the following three exceptions: specialty coffees, coffees with sugar or cream, and hard alcohol with caloric mix), there was a strong correlation between the Beverage Frequency Questionnaire and the 7 day food records in terms of estimated beverage intake [36]. As individuals might have to choose an average container size that may not accurately represent the various sizes consumed for coffee or alcoholic beverages [36].

In a prior study, researchers in Turkey examined the validity and reliability of measurement methodologies for adults' beverage consumption using the beverage intake questionnaire (BIQ) [37]. There was a significant association (*p* < 0.01) between all beverage categories except alcoholic beverage intake when the responses to the food intake record BIQ-1 were examined. Since alcoholic beverages are not consumed in high amounts or frequently in Turkish society, the amount of alcoholic beverages in food consumption records is determined to be less than the BIQ [37]. In addition to underreporting may also occur due to social stigma, particularly regarding alcoholic beverages. Similarly in a study conducted with young Greek individuals, Wilcoxon rank test revealed no significant difference between the beverage frequency questionnaire and 7-day weighted food records, except for 'whiskey/vodka/gin' intake [38]. There was 'low' agreement found for filtered/instant/iced coffee, freshly squeezed orange juice, and some alcoholic beverages in Greek population. Similarly, in our study, low correlations were demonstrated for coffee, fruit juice and alcoholic beverages. The reliability of the Workplace Beverage Intake Questionnaire for total fluid intake and all beverage categories, excluding milk-based beverages and 100% fruit-based beverages, was determined to be moderate [34]. In Rogerson et all.'s study, comparable to our study, low/zero calorie soft drinks, milk-based drinks, and drinks categorized as 'other' (such as powdered drinks prepared with water/milk) exhibited poor reliability. The limited sample size and the low number of individuals who reported consuming drinks in specific categories resulted in uncertainty in the estimates for those categories in our study. In our study as well, a low correlation has been identified for alcoholic beverages. Enhancements in guidance, particularly for the recall of both the size and serving container used for coffee and alcoholic beverages, have been emphasized to improve data accuracy [36]. Although the differences in total sugar beverage and total beverages were not minimal, these differences are similar to other studies, such as Vanderlee et al. The differences between dietary records of total sugary beverages (634 mL) and total beverage intake (241 mL) and BEVQ that we found in our study were similarly identified in the study by Vanderlee et al. [36].

Several limitations were noted in our study. The underrepresentation of males in our sample, resulting in limited gender diversity. Additionally, our sample lacks a wide range of educational backgrounds, further narrowing the variety in this aspect. It is advantageous to assure a more heterogeneous participant pool in future investigations. The predominance of women and university graduates in the sample may limit the generalizability of the findings to the overall Turkish population. The gender imbalance may result in the underrepresentation of men's beverage consumption habits, potentially overlooking gender-based differences. Similarly, the overrepresentation of individuals with higher education levels may prevent the inclusion of diverse socioeconomic groups and their beverage consumption patterns. Future studies should aim to establish a more balanced and representative sample. Increasing diversity in terms of gender, educational background, and socioeconomic status will enhance the applicability and validity of BEVQ-21 across a broader population. Future research should prioritize the inclusion of people who ingested beverages with weaker correlations, such as energy drinks, sweetened fruit drinks and alcoholic beverages. The frequency of alcohol consumption in our country is not very high anyway. Therefore, the correlation levels of beverages with low consumption may have been low. The individuals participating in our study mostly represented the young adult group. Therefore, it would be beneficial to conduct a future study with a wider age range. Furthermore, the one-month duration of the BEVQ and the collection of DR data over a more limited time period may not have accounted for acute and chronic intake of beverage effects and possible changes in beverage intake due to seasonal or monthly variations. Our study was conducted between December and May, and the potential for seasonal variation in beverage consumption (e.g., hot beverages more in winter and cold beverages more in summer) could not be addressed. Future studies examining beverage consumption patterns across seasons would provide a more comprehensive understanding of consumption behaviors. This could have resulted in relatively low correlation coefficients between dietary data and BEVQ-2 in some beverage categories. One of the limitations of our study is that no adjustment was made between the first and second administrations of the BEVQ-21 to reduce possible learning effects. The minor increase in the amounts mentioned in BEVQ-2 compared to BEVQ-1 could potentially be attributed to the learning effect. Future studies may include using longer time intervals between the two administrations or applying alternative question orders to control for learning effects.

Beverages are among the most commonly underreported foods that have a significant impact on energy intake [39]. BEVQ stands out as a significant scale in the assessment of daily energy and total fluid intake in this context. The adaptation of the scale into Turkish and the validation process are crucial for its future use in advanced studies. It has been suggested that taking DR before BEVQ1 may boost the association between DR and BEVQ, as it may lead individuals to be more mindful of eating habits. Our study included the assessment of DR following the administration of BEVQ1, which is one of the notable advantages of our research. Another strength of our research is that market research was undertaken in the course of assessing the energy composition of beverages. Country to country, beverages may have varying energy contents. To this end, the energy content of beverage groups were calculated using the mean value derived from the label information of at least three brands of beverages included in that beverage category.

## Conclusion

The updated Turkish version of the BEVQ-15 offers a nutritional evaluation instrument capable of efficiently appraising overall beverage intake as well as the intake of several beverage categories. This survey, incorporates modifications tailored to the cultural context of Turkey. The BEVQ-21 added additional beverages such as plain mineral water, flavored mineral water, kefir, and turnip, which are consumed in Turkish society but were not originally included in the BEVQ-15 questionnaire. Furthermore, although tea and coffee were collectively examined in BEVQ-15, the BEVQ-21 distinguished between black tea and coffee, which are commonly consumed in contemporary society, and herbal teas. This can be utilized in future research on the topic of hydration and beverage intake in adults. Conducting additional validation studies in different populations, such as children or older adults and clinical populations with chronic diseases, will help assess the broader applicability of BEVQ-21 across various demographic groups in Turkey. This will enable the BEVQ-21 to be more widely applicable in public health and clinical settings in our country. Additionally, there may be an intention to create a mobile application for the purpose of gathering data about beverage consumption. The potential use of a mobile application for future data collection could provide real-time data entry, helping participants record their beverage consumption more accurately and reducing recall bias. Additionally, user-friendly interfaces, reminder notifications, and visual guides could enhance participant engagement and improve data quality. As a result, it is anticipated that BEVQ-21 will be beneficial for assessing total beverage intake and determining energy intake from beverages in future studies. Considering that energy intake from beverages can contribute to the prevalence of obesity, we believe that accurately determining beverage consumption is crucial in our study. If beverage consumption is not thoroughly assessed by DR, underestimation of energy intake may occur. Therefore, the combined use of the BEVQ-21, which provides a detailed evaluation of beverage consumption, and DR is expected to contribute significantly to future studies. The potential use of BEVQ-21 alongside dietary intake records in future nutritional research, obesity studies, and clinical nutrition practices in Turkey is also considered. Specifically, evaluating beverage consumption habits can help in understanding overall dietary patterns, monitoring energy intake, and developing effective policies for the prevention and management of nutrition-related diseases. Moreover, it can support the development of personalized nutrition interventions tailored to individual needs.

## Data Availability

No datasets were generated or analysed during the current study.
